# Causal network localization of brain stimulation targets for trait anxiety

**DOI:** 10.21203/rs.3.rs-4221074/v1

**Published:** 2024-04-09

**Authors:** Shan H. Siddiqi, Julian Klingbeil, Ryan Webler, Ian H. Kratter, Daniel M. Blumberger, Michael D. Fox, Mark S. George, Jordan H. Grafman, Alvaro Pascual-Leone, Andrew R. Pines, R. Mark Richardson, Pratik Talati, Fidel Vila-Rodriguez, Jonathan Downar, Tamara Hershey, Kevin J. Black

**Affiliations:** 1. Center for Brain Circuit Therapeutics, Brigham & Women’s Hospital, Boston, MA; 2. Department of Psychiatry, Harvard Medical School; 3. Department of Neurology, University of Leipzig Medical Center; 4. Department of Psychiatry and Behavioral Sciences, Stanford University School of Medicine; 5. Department of Psychiatry, University of Toronto; 6. Temerty Centre for Therapeutic Brain Intervention, Centre for Addiction and Mental Health, Toronto, ON; 7. Department of Psychiatry, Medical University of South Carolina; 8. Ralph H. Johnson Veterans Affairs Hospital; 9. Shirley Ryan AbilityLab; 10. Northwestern University Feinberg School of Medicine; 11. Department of Neurology, Harvard Medical School, Boston, MA, USA; 12. Hinda and Arthur Marcus Institute for Aging Research; Deanna and Sidney Wolk Center for Memory Health, Hebrew SeniorLife, Boston, MA, USA; 13. Department of Neurosurgery, Massachusetts General Hospital; 14. Department of Neurosurgery, Harvard Medical School; 15. Non-Invasive Neurostimulation Therapies Laboratory, Department of Psychiatry and School of Biomedical Engineering, University of British Columbia; 16. Departments of Psychiatry, Radiology, Neurology and Neuroscience, Washington University School of Medicine

## Abstract

Transcranial magnetic stimulation (TMS) and deep brain stimulation (DBS) can treat some neuropsychiatric disorders, but there is no consensus approach for identifying new targets. We localized causal circuit-based targets for anxiety that converged across multiple natural experiments. Lesions (n=451) and TMS sites (n=111) that modify anxiety mapped to a common normative brain circuit (r=0.68, p=0.01). In an independent dataset (n=300), individualized TMS site connectivity to this circuit predicted anxiety change (p=0.02). Subthalamic DBS sites overlapping the circuit caused more anxiety (n=74, p=0.006), thus demonstrating a network-level effect, as the circuit was derived without any subthalamic sites. The circuit was specific to trait versus state anxiety in datasets that measured both (p=0.003). Broadly, this illustrates a pathway for discovering novel circuit-based targets across neuropsychiatric disorders.

## One-Sentence Summary

A causal brain circuit for anxiety can act as a therapeutic target, charting a path for discovering targets across disorders.

## Introduction

Anxiety disorders are the most common class of mental illness and the world’s sixth-leading cause of disability^1^, but they remain underdiagnosed and undertreated^2^. Despite recent advancements in rapid-acting antidepressants, no new treatments for anxiety disorders have been approved by the US Food and Drug Administration (FDA) in the last 15 years^3^. For patients who do not remit with cognitive-behavioral therapy and serotonergic antidepressants, few safe and effective alternatives remain^3^. Transcranial magnetic stimulation (TMS) and deep brain stimulation (DBS) are therapeutic brain stimulation tools that have shown promise for major depressive disorder (MDD) and obsessive-compulsive disorder, and may improve anxiety in a subset of these patients^4^. Preliminary studies have shown that anxiety changes induced by TMS^5^ and DBS^6,7^ depend on targeting specific brain circuits. However, optimal circuit-based targets have not been definitively localized or characterized. It also remains uncertain if targeting should focus on trait anxiety, the chronic predisposition towards anxiety, or state anxiety, the acute autonomic experience.

Brain stimulation targets for specific symptoms may be identified by mapping brain circuitry that is causally implicated in that symptom^8^. For instance, using a normative connectome database (n=1000)^9^, we recently mapped a common causal network for depression based on connectivity of lesions that cause depression, TMS sites that relieve depression, and DBS sites that cause or relieve depression^10^. Connectivity of TMS and DBS sites to this convergent circuit was a better out-of-sample predictor of antidepressant response than other candidate regions such as the subgenual cingulate^10^. Based on this principle, brain lesion data have also been used to optimize stimulation sites for parkinsonism^10^, addiction^11^, tics^12^, epilepsy^13^, pain^14^, and tremor^15^.

In each of these cases, lesion-derived circuits were used to explain variability in outcomes with stimulation targets that were already in clinical use. If this approach successfully extends to anxiety, it would demonstrate that lesion localization can also be used to discover novel brain stimulation targets. However, because anxiety and depression often covary, mapping anxiety-specific circuits may require disentangling comorbid depression^5,16^. Pilot work suggests that anxious/somatic versus dysphoric symptoms^5^ in MDD can be modulated by distinct circuit-based TMS targets, but it remains unclear if this generalizes. The present study mapped a more robust transdiagnostic anxiety network using a hypothesis-driven approach with validated anxiety scales using natural experiments in multiple settings across multiple clinical disorders. Aphoristically, multimodal convergence enables strong causal inference about brain circuitry, as different natural experiments can compensate for each other’s confounders^8^. Pragmatically, this convergence demonstrates that stimulation targets can be identified using lesion data, which are widely available given the high prevalence of stroke and brain injury.

## Results

### Characteristics of included datasets

We identified seven datasets (n=936) in which anxiety and depression were measured after TMS, DBS, or lesions to different brain regions. The datasets were categorized into four groups: incidental lesions, scalp-based TMS targets with incidental variability in location, MRI-guided TMS targets with incidental variability in connectivity, and incidental DBS sites (Table 1). Datasets are described further in Appendix S1. Our overarching hypothesis was that these four categories would converge on common circuitry that is causally involved in anxiety, independent of depression. Across all datasets, the weighted mean correlation between anxiety and depression was r = 0.53, so 72% of the variance in anxiety was not explained by depression.

### TMS and lesions converge on common causal circuitry

**Figure 1:**
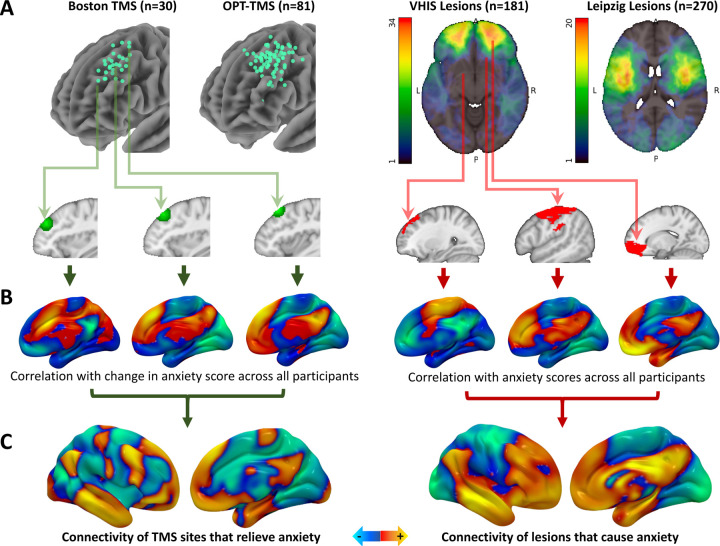
Lesion and TMS network mapping. **(A)** 111 TMS sites and 451 brain lesions were localized (localization methods listed in Table 1). Three representative examples are highlighted here. **(B)** A normative fcMRI connectome database (n=1000) was used to estimate the whole-brain connectivity of each TMS site and lesion. These connectivity profiles were compared with anxiety outcomes across all patients, controlling for depression. **(C)** For each dataset, this yielded the connectivity of TMS sites or lesions that causally modify anxiety.

As in our prior work on depression^10^, we hypothesized that normative connectivity of high-frequency TMS sites (which are believed to increase excitability) that relieve anxiety would be similar to normative connectivity of lesions (presumably inhibitory) that cause anxiety. TMS sites varied markedly across the left DLPFC, while lesions were distributed across different parts of the brain (Fig. 1a). For each TMS site or lesion, whole-brain functional connectivity was estimated using a large-scale fcMRI connectome database (n=1000) (Fig. 1b), which was treated as a normative wiring diagram. These connectivity profiles were compared with clinical outcomes to identify the connectivity of TMS sites or lesions associated with anxiety (Fig. 1c). For TMS, the clinical outcome was change in the “anxiosomatic” symptom cluster score as defined in our prior work^5^. Of note, this score was defined using item-level clustering rather than a validated anxiety scale, so it was compared against multiple other datasets that used validated anxiety scales. For lesions, the clinical outcome was post-lesion anxiety severity. Pre-lesion anxiety scores were not collected because lesions were unexpected, so the analysis assumes that pre-lesion anxiety is randomly distributed with respect to lesion location. Depression score was included as a covariate in all cohorts. In the lesion cohorts, lesion size was also included as a covariate.

**Figure 2:**
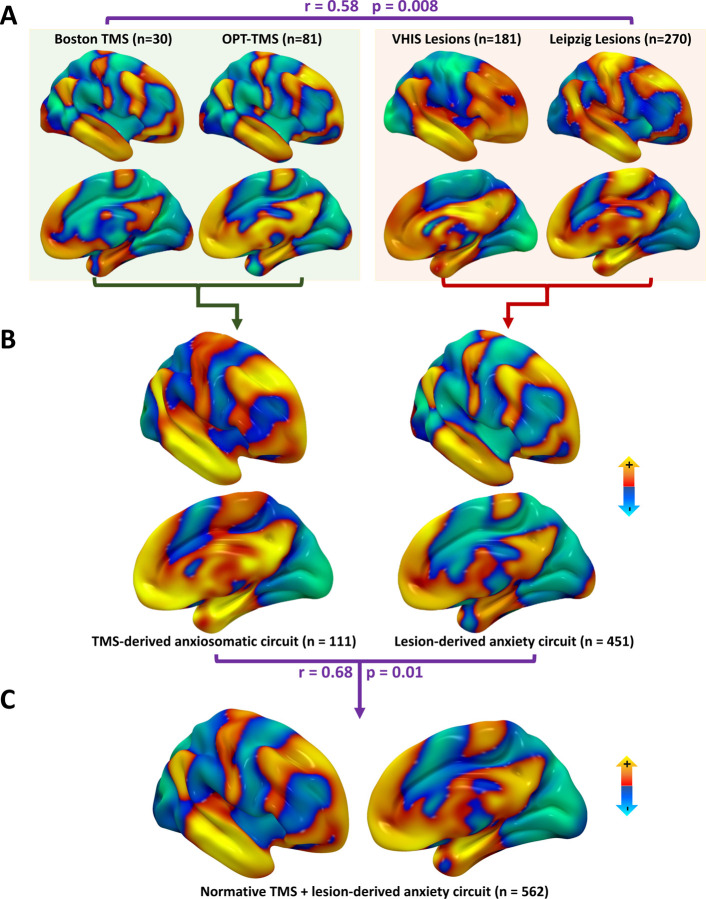
TMS and Lesions converge on similar circuitry for anxiety. Warm colors represent connectivity of TMS sites that relieve anxiety or lesions that cause anxiety, and cool colors are the converse. **(A)** The two TMS datasets and the two lesion datasets yielded circuit maps that were significantly similar to each other (spatial r = 0.57, p = 0.008). **(B)** The overall TMS network map and the overall lesion network map were significantly similar to each other (spatial r = 0.68, p = 0.01). **(C)** The TMS and lesion-derived maps circuits were combined into a single anxiety circuit.

This yielded a circuit map for each of the four datasets (Fig. 2a). These four maps showed high spatial cross-correlation (r=0.58) which was significantly stronger than chance (p=0.008) on permutation testing in which each patient’s clinical outcome was randomly shuffled with a different patient’s neuroimaging, as in our prior work^10^. The circuit maps were then combined into a weighted mean map for each modality (Fig. 2b). The combined TMS network map was significantly similar to the combined lesion network map (r=0.68, p=0.01). These maps were combined into a single TMS and lesion-derived anxiety circuit (Fig. 2c).

### Individual variability in the anxiety circuit

**Figure 3:**
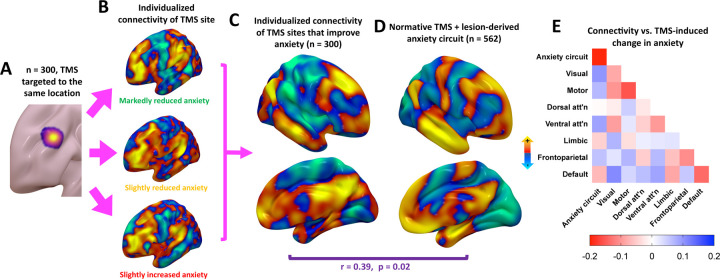
A similar anxiety circuit derived from individualized connectivity. **(A)** 300 patients with MDD received TMS targeted to the same coordinate using MRI neuronavigation. **(B)** Even with identical targets, individualized fcMRI yielded different connectivity patterns of this target in different patients. Three representative examples are depicted here. **(C)** A TMS network map for anxiety was mapped based on variability in individualized connectivity of the common TMS site. **(D)** The TMS network map derived from individualized connectivity was significantly similar to a convergent TMS/lesion network map derived from normative connectivity in independent datasets. **€** TMS-induced change in anxiety was predicted by baseline connectivity within the anxiety circuit, but not other circuits.

Next, we studied 300 patients with MDD who completed fcMRI before a course of TMS targeted to a specific MNI coordinate (−38, 44, 26) in the DLPFC. Because the target was held constant (Fig. 3a), this isolates the effect of variability in functional connectivity to the anxiety circuit. We computed an anxiety circuit (controlling for depression) using the methods above, but using individualized rather than normative connectivity (Fig. 3b). This yielded a whole-brain map representing individualized connectivity of TMS sites that modify anxiety (Fig. 3c), which was significantly similar to the normative anxiety circuit described above (Fig. 3d) (spatial r=0.39, p=0.02). TMS site connectivity to the anxiety circuit predicted TMS-induced change in anxiety, controlling for depression (r=0.14, p=0.02).

In this dataset, we found a high incidence of increase in anxiety after TMS (n=59, 20%), which was surprising given that prior TMS clinical trials have not reported anxiety as a common side effect^25,26^. We hypothesized that this may be because most patients’ TMS sites were negatively connected to the anxiety circuit (mean r= −0.19 ± 0.13, p<0.001), while prior trials targeted regions that tend to be positively connected to the anxiety circuit^5^. We tested this hypothesis by binarizing patients according to whether anxiety scores increased by at least one point after treatment. TMS sites that worsened anxiety showed stronger negative connectivity to the anxiety circuit, whether controlling for depression (p=0.002) or not (p=0.007). In a combined regression model, TMS site connectivity to the anxiety circuit was independently associated with categorical worsening in anxiety (p=0.02), but not continuous change in anxiety (p=0.54). Thus, TMS sites negatively connected to the anxiety network led to worsening in anxiety.

To explore clinical significance, we repeated the analysis with two alternative binarization cutoffs: a 3-point increase or a 3-point decrease in anxiety score. This 3-point threshold (0.5 points per item) was equal to the median TMS-induced change and is consistent with clinically meaningful changes reported previously^27,28^. In both conditions, direction of anxiety change was still associated with TMS site connectivity to the anxiety circuit (p=0.01 and p=0.02, respectively). This effect was detectable only in patients who had comorbid anxiety disorders at baseline (n=173, r=0.19, p=0.01), but not in those who did not have baseline anxiety (n=127, r=0.08, p=0.38). Thus, the effect of TMS sites on anxiety was driven by clinically meaningful changes in patients with clinically significant baseline symptoms, consistent with prior DBS studies^24^.

Baseline connectivity within the anxiety circuit also predicted TMS-induced change in anxiety (r= −0.20, p=0.0007). Again, this was driven by categorical worsening in anxiety (p=0.003) rather than continuous change (p=0.21). Behaviorally, this was also specific to anxiety (r= −0.14, p=0.01) relative to depression (r=0.07, p=0.20). Anatomically, this was specific to the anxiety circuit relative to seven control networks as defined by Yeo et al., 2011 (Fig. 3e). Default mode network (DMN) connectivity also predicted anxiety change, but the anxiety network was a stronger predictor than the DMN (p=0.025).

We also explored the alternative possibility that this result was related to association between our anxiety circuit and baseline anxiety. If true, this could create a spurious association between the circuit and treatment-induced change in anxiety. Baseline anxiety was not significantly associated with TMS site connectivity to the anxiety circuit (r = −0.02, p=0.69) or connectivity within the anxiety circuit (r= −0.09, p=0.13).

### Creation of a convergent causal anxiety circuit

**Figure 4:**
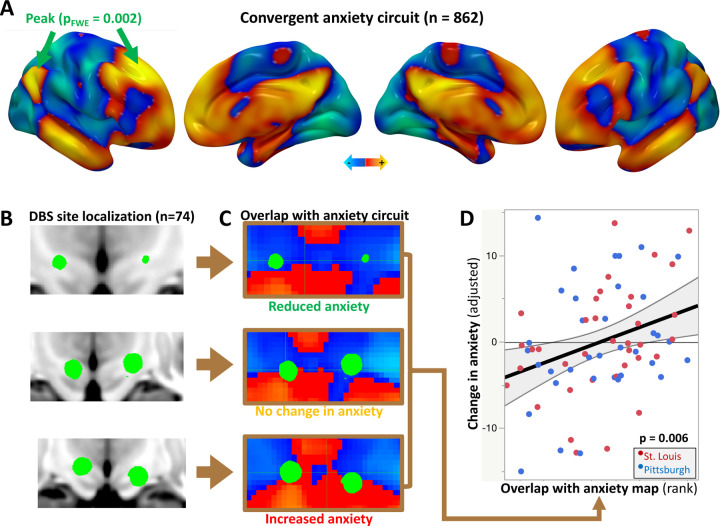
Validation of the convergent anxiety circuit. **(A)** All lesion and TMS-derived anxiety datasets were combined into a convergent weighted mean anxiety circuit. The peaks in this circuit, in the right superior frontal gyrus and lateral parietal lobe, were significantly stronger than chance on permutation testing. **(B)** 74 bilateral DBS electrodes were localized across two independent cohorts. Three representative examples are depicted here. **(C)** For each DBS site, overlap with the anxiety circuit was quantified and compared with anxiety outcome. **(D)** DBS site overlap with the anxiety circuit was associated with change in anxiety, controlling for baseline anxiety and depression (p=0.006).

We combined the anxiety circuit maps from the above five datasets into a weighted mean causal anxiety circuit (Fig. 4a), following our prior recommended approach for causal brain mapping^8^. Convergence strengthens causal inference because the confounders from each modality are mitigated by other modalities with different confounders^8^. The peaks in this circuit were in the right superior frontal gyrus (SFG), MNI coordinate (22, 31, 42), and the right lateral parietal lobe (LPL), MNI coordinate (−47, −59, 32). Both peaks were significantly stronger than chance using voxel-wise permutation testing (p_FWE_=0.002 in both cases)^29^.

### External validation of the anxiety circuit using DBS

To evaluate generalizability of our approach, we validated the TMS- and lesion-derived anxiety circuit against a distinct stimulation modality with a markedly different anatomical target. We achieved this using DBS of the subthalamic nucleus (STN) in Parkinson’s disease (Fig. 4b). None of the TMS sites and only one of the lesions touched the STN, so the topography of our circuit in the STN is driven by connectivity to distant sites. Across two datasets (total n=74), DBS site overlap with the anxiety circuit (Fig. 4c) predicted DBS-induced change in anxiety, independent of depression (r=0.32, p=0.006) (Fig. 4d). Of note, overlap was calculated separately for the left and right stimulation sites, and then added together.

### Comparison to alternative models

We benchmarked our anxiety circuit (derived from causal lesion and TMS data) against other approaches for identifying therapeutic targets. Alternative approaches for identifying brain stimulation targets include (1) correlative neuroimaging, (2) lesion mapping without network analysis, and (3) incidental detection of useful neuromodulation targets through clinical experience, (4) location of receptors for medications used to treat anxiety (serotonin transporter or GABA_A_ receptors).

We attempted to predict TMS- and DBS-induced change in anxiety based on stimulation site connectivity to regions of interest (ROIs) derived from these four approaches. To model (1) correlative neuroimaging, we derived ROIs from two sources: a recent published meta-analysis of neuroimaging in anxiety disorders^30^ and an automated meta-analysis of neuroimaging findings associated with the search term “anxiety” using NeuroSynth^31^. NeuroSynth was also used to generate comparators using search terms associated with other psychiatric disorders. For (2) lesion mapping without network analysis, we conducted voxel lesion symptom mapping^32^ in the 451 lesions described above. To model (3) targets in current clinical use, we generated ROIs at the approximate location of common TMS sites in the right and left DLPFC, locations that are commonly targeted with TMS for anxiety. We also modeled the H1 and H7 TMS coils, the first TMS coils to receive FDA clearance for anxious depression and obsessive-compulsive disorder, respectively^4,33,34^. To localize (4) pharmacotherapeutic targets for anxiety, we employed published ROIs of various neurotransmitter receptor distributions, including serotonin transporters and GABA_A_ receptors^35^. Altogether, this yielded 64 ROIs.

Our anxiety circuit predicted outcomes in each brain stimulation cohort better than any of these 64 ROIs (Table S1). Of note, the anxiety circuit in each case was re-generated after excluding the modality that was being predicted, following the same approach that was used for the above analyses. For normative TMS datasets and the individualized TMS dataset, the second-best predictor was the right 5 cm target, a commonly-used TMS target for anxiety. For the DBS datasets, the second-best predictor was the distribution map of the serotonin transporter, which is targeted by selective serotonin reuptake inhibitors, the standard first-line pharmacotherapy for anxiety disorders.

### Specificity to trait anxiety

**Figure 5:**
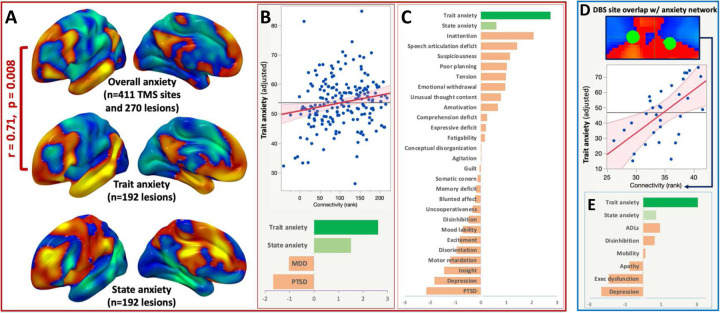
**Specificity to trait anxiety. (a)** TMS sites and Cologne lesions that modify anxiety were connected to a similar circuit to VHIS lesions that specifically modify trait anxiety, but not state anxiety. **(b)** In the VHIS dataset, lesion connectivity to the out-of-sample anxiety circuit was significantly associated with trait anxiety, independent of trait anxiety, MDD, or PTSD. When expanding this specificity analysis to all items on the neurobehavioral rating scale **(c)**, the out-of-sample anxiety circuit was more associated with trait anxiety than state anxiety or 26 control measures. **(d)** In the St. Louis dataset, DBS site overlap with the overall anxiety circuit was associated with trait anxiety. **€** This association was specific to trait anxiety, but not state anxiety or six other control measures associated with Parkinson’s disease and other neurodegenerative disorders.

We compared trait and state anxiety using two datasets that collected the Spielberger state-trait anxiety inventory. First, in the VHIS dataset, we repeated the circuit mapping procedure, but used trait anxiety as the primary outcome, controlling for state anxiety, MDD diagnosis, and PTSD diagnosis. The resulting trait anxiety circuit in this dataset was similar to a leave-one-dataset-out anxiety circuit (spatial r=0.71, p=0.008) (Fig. 5a). Similar results were not observed for state anxiety (r= −0.28). Lesion connectivity to the anxiety circuit was significantly associated with trait anxiety (r=0.22, p=0.003), independently of state anxiety, MDD, or PTSD (Fig. 5b). These results remained unchanged when removing MDD as a covariate (p=0.01), removing PTSD (p=0.04), adding dysthymic disorder (p=0.003), using continuous measures of depression and PTSD (BDI and Clinician Assessment for PTSD Symptoms) instead of categorical diagnosis (p=0.005), or using total neurobehavioral rating scale (NBRS) score as a covariate (p=0.01). Our anxiety circuit was also specific to trait anxiety relative to 26 other control measures from the NBRS (p=0.007) (Fig. 5c).

Next, we examined trait anxiety in the St. Louis dataset (Fig. 5d). DBS site overlap with the anxiety circuit predicted post-DBS trait anxiety (r = 0.48, p = 0.003), independent of baseline trait anxiety and pre/post activities of daily living (ADLs), disinhibition, mobility, apathy, executive dysfunction, and depression (Fig. 5e). This result was not driven by any individual covariate, as it remained significant even when removing each individual comorbid symptom, all comorbid symptoms, or all pre-DBS scores from the model (p<0.05).

### Application to baseline anxiety disorders

Presence of comorbid anxiety disorders at baseline was recorded in the OPT-TMS dataset (n=20), the THREE-D dataset (n=173), and the St. Louis DBS dataset (n=17). Treatment-induced change in anxiety was predicted by stimulation site connectivity to a leave-one-dataset-out anxiety circuit both in patients with baseline anxiety disorders (r=0.19, p=0.006) and without (r=0.15, p=0.03).

## Discussion

Across seven datasets including 936 individuals, we derived and validated a brain circuit for transdiagnostic anxiety despite multiple sources of heterogeneity between datasets, which would bias us against detecting a common substrate. Convergence across multiple modalities strengthens causal inference, as each modality has different confounders that compensate for each other^8^. Our localization builds on prior cross-species models of anxiety. Human studies have characterized trait anxiety as a transdiagnostic deficit of mPFC control over emotional circuitry, but correlative neuroimaging is of uncertain significance for targeting a causal treatment^36–38^. Rodent studies have causally localized fear extinction to the ventral mPFC, but have not definitively translated this into anxiety in humans^39^. Our results unite these human and animal models, and raise several implications regarding future target discovery across disorders, personalization of targets, anxiety as a side effect of stimulation, and trait versus state anxiety.

First, convergence across lesions and stimulation demonstrates that lesion localization can reveal new therapeutic targets. Prior studies have validated lesion network mapping against existing FDA-cleared TMS and DBS targets for depression^10^, addiction^11^, Parkinson’s disease^10^, tremor^15^, and epilepsy^13^. Other studies have applied lesion network localization to a wide range of brain functions, from hallucinations to free will^40^. Using anxiety as an example, our results suggest that lesions can be used to identify novel circuit-based stimulation targets that have not yet been tested in randomized controlled trials.

Second, our results point to a novel brain stimulation target for anxiety in the right superior frontal gyrus (sFG), extending through the medial prefrontal cortex (mPFC) and the posterior dorsolateral prefrontal cortex (dlPFC). A recent TMS clinical trial incidentally tested this hypothesis by comparing the “5.5 cm” target, which partly overlaps with the positive part of our anxiety circuit^5^, to the “Beam F3” target, which only overlaps with the negative part of our anxiety circuit^5^. As expected, the 5.5 cm target was significantly more effective for anxiety, independent of depression^16^. Of note, these regions overlap broadly with the default mode network (DMN), which has been extensively implicated in rumination, a core component of trait anxiety^41–43^. However, the circuit also predicted anxiety after DBS to the STN, which is not part of the DMN. Furthermore, connectivity within the circuit outperformed the DMN for predicting TMS-induced anxiety change. The circuit also outperformed multiple other brain regions that have previously been targeted for anxiety treatment.

Third, in a cohort where all patients received TMS at the same cortical location, individualized connectivity of the TMS site to the anxiety circuit explained variability in treatment-induced change in anxiety. This suggests potential clinical value for personalizing the circuit. Individualized connectivity can be used to optimize TMS targets for depression^21,44–48^ and memory^49,50^, but there remains debate about whether it is adequately useful to implement on a large scale^51^. Although we observed a small effect size of personalization, this may be due to older imaging parameters with low spatial resolution and low sampling duration; recent advances in precision individualized fMRI can markedly improve signal quality^52^. These advances may argue in favor of fMRI-guided brain stimulation.

Fourth, while some TMS and DBS targets reduced anxiety across multiple disorders, others worsened pre-existing anxiety. This supports the conceptualization of TMS and DBS as tools, not as treatments – if the tools are used incorrectly, they can be counterproductive. This is consistent with studies in which some TMS targets appeared to exacerbate anxiety in patients with posttraumatic stress disorder^53^, patients with arachnophobia^54^, and healthy volunteers^55^. Assessment of TMS effects on anxiety may be clouded by heterogeneous targeting and by improvement in comorbid depression, implying that anxiety may be under-recognized as a side effect of TMS. This finding unmasks a clinical quandary, as the targets that worsen anxiety were similar to those that are believed to be most effective for depression^17^. This is consistent with our prior work showing that target circuits for dysphoric versus anxiosomatic symptom clusters are anti-correlated with each other^5^, and may explain why optimized TMS targets for depression have not markedly improved clinical outcomes.

Fifth, our results were specific to trait versus state anxiety, consistent with prior studies implicating the sFG in trait anxiety in healthy individuals^42^. Trait anxiety, or the chronic predisposition towards anxiety, is a transdiagnostic component of many psychiatric disorders^56^ and other conditions such as chronic pain^57^. By contrast, state anxiety refers to transient autonomic arousal in response to stressful events, which can be a normal phenomenon. This distinction suggests that our anxiety circuit is driven by changes in chronic underpinnings of anxiety rather than transient responses to stimuli. This may explain why the trait anxiety circuit did not prominently feature the amygdala or insula, which are consistently implicated in studies of state anxiety^58^. Rather, it overlapped largely with the default mode network, which has been consistently implicated in trait rumination^43^. Of note, while we used the classical state-trait model in this study, future studies may incorporate more recent models that include more precise subtyping of anxiety phenotypes.

While the multi-dataset and multi-modal analysis is a strength of this study, this entails the inherent limitations of a natural experiment with heterogeneous datasets. Each dataset had its own unique confounders and potential sources of noise, thus limiting the effect size that can be detected by this method. For instance, pre-lesion outcomes were not collected, thus necessitating the assumption that pre-lesion anxiety was randomly distributed with respect to lesion location; while this assumption may be reasonable, it introduces noise into the analysis. Variable data quality may also introduce noise; for instance, the OPT-TMS dataset used MRI fiducial markers rather than neuronavigated TMS site localization, while the THREE-D dataset employed fcMRI with low spatial resolution and short sampling duration. By contrast, the St. Louis DBS dataset used image-guided lead localization whose accuracy was validated on autopsy^59^. This heterogeneity biases us in favor of a negative result, so our positive results suggest that the large sample size provided enough signal to offset the noise. To prevent further heterogeneity, we limited the lesion-based analysis to predominantly grey matter lesions; future work may also attempt network localization using leukodystrophies, hypertensive leukoaraiosis, or multiple sclerosis. The use of subjective anxiety scales presents strong face validity for relevance to clinical syndromes but limits our ability to assess specific cognitive processes. Future studies could index how manipulation of our circuit affects anxiety-relevant processes such as fear extinction (31) and emotional conflict resolution (28). Finally, our anxiety circuit was derived from a transdiagnostic sample with varying levels of anxiety. Importantly, TMS and DBS site connectivity to our circuit predicted anxiety change in patients with anxiety disorders. Nevertheless, prospective clinical trials in anxiety disorder samples are necessary.

Overall, we used multiple natural experiments to demonstrate the existence of a brain circuit that is causally involved in trait anxiety across multiple natural experiments, uniting prior human and animal models. The circuit contained a peak in the right superior frontal gyrus which we propose as a new brain stimulation target for anxiety. Our transdiagnostic approach lays out a path for discovery of new brain stimulation targets across neuropsychiatric disorders.

## Methods

### Data inclusion

Datasets were identified that collected validated anxiety and depression scales along with a causal neuronal circuit manipulation, such as penetrating head trauma, stroke, TMS, or STN DBS. Datasets were separated into four categories based on similar data types. First, two datasets (n=111) included TMS applied to the left dorsolateral prefrontal cortex (DLPFC) using scalp landmarks, leading to incidental inter-individual variability in cortical stimulation site. Second, two datasets included brain lesions from stroke (n=270) or penetrating head trauma (n=181) to different locations in the brain. Third, one dataset (n=300) included TMS applied using structural neuronavigation, so all participants were stimulated at the same cortical location, but individual functional connectivity MRI (fcMRI) was used to detect inter-individual variability in the circuit that was incidentally stimulated. This dataset was analyzed separately from the remaining TMS data because individualized functional connectivity cannot be assumed to provide the same information as normative connectivity. Fourth, two datasets (n=76) included patients with Parkinson’s disease who received DBS to bilateral subthalamic nuclei (STN) based on intraoperative testing. This dataset was also analyzed separately because it cannot be assumed that bilateral STN DBS sites can be combined into a single functional connectivity seed.

For the two datasets used to derive our previously-published anxiosomatic circuit (Boston TMS and OPT-TMS), the anxiosomatic symptom cluster was treated as the anxiety scale, while the dysphoric cluster was treated as the depression scale. All datasets included MRI or CT based localization of the lesion or stimulation site. Participants provided written informed consent with institutional review board approval or a waiver of consent was approved for retrospective research. Dataset characteristics are summarized in Table 1.

For each analysis, patients with missing data for the relevant metrics were excluded. Each dataset was analyzed independently to prevent idiosyncratic characteristics of one dataset from influencing results in a different dataset. When different datasets were categorized together, their results were combined using a weighted mean.

No *a priori* power analysis was conducted, but to our knowledge, this is the largest study of lesion, TMS, and DBS localization for any disorder.

### Generation of circuit maps by dataset

Each participant’s lesion or stimulation site was localized onto a standard Montreal Neurological Institute brain atlas. TMS sites were modeled as a decaying sphere with maximal radius of 12mm, as in our prior work. A normative connectome database (n=1000) was then used to estimate the whole-brain connectivity of the lesion or TMS site, as in our prior work. Within each dataset, these connectivity maps were compared with anxiety outcome using voxel-wise partial correlation, controlling for depression. For lesions, the outcome was defined as severity of anxiety and depression, with lesion size as an additional covariate. For stimulation, the outcome was defined as change in anxiety and depression, with baseline severity as an additional covariate. This analysis yielded a circuit map for each dataset representing the connectivity of lesions or stimulation sites that modify anxiety, independent of depression.

### Comparing normative circuits derived from TMS and lesions

First, we tested the hypothesis that lesions and TMS converge on the same normative circuit. The Boston TMS and the OPT-TMS maps were combined into an overall normative TMS network map, while the VHIS lesion and Cologne lesion maps were combined into an overall lesion network map. These two combined maps were compared with one another using spatial correlation. To confirm if this spatial correlation was stronger than chance, we designed a permutation test in which the spatial correlation was recomputed 10,000 times after randomly re-assigning each patient’s clinical outcome to a different patient’s neuroimaging, as in our prior work. A p-value was computed as the percentage of cases in which the real spatial correlation was stronger than the permuted spatial correlation. To ensure that the results were not driven by the categorization step, we also computed the mean spatial cross-correlation between all four circuit maps, and again used permutation testing to confirm if this is stronger than chance. We then combined all four maps into a single weighted mean normative anxiety circuit.

### Individualizing the network

Next, we tested the hypothesis that individualizing the anxiety network can explain additional variance in clinical outcomes. In the THREE-D TMS dataset, all participants were stimulated at the same MNI coordinate, thus eliminating the effect of individual variability in stimulation location. All participants also completed baseline resting-state fMRI scans, enabling assessment of individualized connectivity in a setting where stimulation location is held constant. We computed whole-brain connectivity of each participant’s stimulation site and compared it to the normative anxiety circuit using spatial correlations. This value was used as an estimate of TMS site connectivity to the anxiety circuit. We used this baseline value to predict TMS-induced change in anxiety, controlling for TMS-induced change in depression.

We also generated a whole-brain anxiety network in this dataset using the same methods as above but replacing normative connectivity with individualized connectivity (Fig. 3b). This yielded a whole-brain circuit representing the individualized connectivity of stimulation sites that selectively modify anxiety. This network was compared with the normative anxiety circuit using spatial correlations with permutation testing as above.

To test if connectivity within this circuit was associated with clinical outcomes irrespective of the stimulation site, we measured seed-based connectivity within the network as in our prior work. We compared this to seed-based connectivity within and between 7 control networks as defined by Yeo et al. We hypothesized that TMS-induced change in anxiety would be specifically associated with baseline connectivity within our anxiety network.

### Creation of a convergent causal anxiety circuit

We combined all the individualized and normative anxiety circuits into a single overall convergent causal anxiety circuit using weighted means. We localized the center of gravity of the two strongest voxel clusters in this circuit using FSL’s cluster algorithm. To determine if these peaks were stronger than chance, we recomputed the convergent circuit after randomly permuting each patient’s imaging with a different patient’s clinical outcomes. We computed an FWE-corrected p-value as the percentage of cases in which the real peak was stronger than the peak in the permuted map, as in prior work^29^.

### External validation using DBS

As an external validation of the anxiety circuit, we tested it against the locations of STN DBS sites in patients with Parkinson’s disease. This dataset was used as an external validation rather than using it to generate a circuit map because each patient had bilateral stimulation sites, unlike all the other datasets in which patients had a single stimulation site or lesion. By quantifying overlap of each DBS site with the anxiety circuit, we were able to test each site independently, while generating a circuit map would require combining them both into a single region of interest, which may discard valuable information. Each DBS site was localized as in the original study in which it was acquired. In the St. Louis dataset^24^, DBS sites were localized on postoperative head CT using an automated algorithm^60^ which has been validated against autopsy results^39^. In the Pittsburgh dataset, DBS sites were localized using manual registration in LEAD-DBS with individualized modeling of the volume of activated tissue^23^. For each DBS site, we quantified overlap by computing the mean voxel value of the anxiety circuit that falls within the DBS field, and then added the values for the left and right sides. We compared this overlap metric to DBS-induced change in anxiety as measured by the STAI, controlling for baseline STAI, change in BDI, and baseline BDI. To confirm that these results were not driven by overall improvement in Parkinson’s disease, we also repeated this analysis after controlling for change in motor symptoms.

### Alternative models

Alternative approaches for treating anxiety were identified based on multiple approaches. 64 regions of interest (ROIs) were selected as described above. We then computed TMS site connectivity or DBS site overlap with these ROIs, following the same methods as in the earlier analysis with the anxiety circuit. We hypothesized that stimulation site connectivity/overlap with the anxiety circuit would be predict stimulation-induced change in anxiety better than any of the alternative ROIs.

### Specificity analysis

Because our data were longitudinal, we hypothesized that the anxiety circuit was associated with trait anxiety (more likely to be chronic) rather than state anxiety (more likely to be acute). We tested this in two datasets that measured trait and state anxiety separately – the VHIS lesion dataset and the St. Louis DBS dataset. In the VHIS dataset, we repeated the above methods to generate a circuit map for trait anxiety, independent of state anxiety. We compared this circuit to an overall anxiety circuit generated from the remaining data using spatial correlations with permutation testing. We also repeated this analysis for state anxiety rather than trait anxiety.

We further assessed specificity against other metrics in two separate but related analyses. First, we computed a linear model to predict lesion connectivity to the anxiety circuit based on four independent predictors: trait anxiety, state anxiety, MDD diagnosis, and PTSD diagnosis. Second, we repeated this analysis using 24 additional predictors based on individual behaviors measured using the neurobehavioral rating scale (NBRS), as in our prior work. MDD was not included as a covariate in this analysis because the NBRS includes a depression item. The anxiety item in the NBRS was excluded because trait and state anxiety were already present in the model. The hallucinations item in the NBRS was excluded because no participants reported even mild hallucinations. Of note, the NBRS anxiety item was excluded from this analysis because it overlaps with state and trait anxiety, and the hallucinations item was excluded because no participants had mild, moderate, or severe hallucinations. The remaining items had at least 15 participants with positive scores.

We repeated a similar analysis in the St. Louis DBS dataset. In a linear model, we predicted DBS site overlap with the anxiety circuit based on trait anxiety, state anxiety, and several control symptoms relevant to Parkinson’s disease and neurodegenerative disorders. These control metrics included activities of daily living and mobility as measured by the Parkinson’s disease questionnaire (PDQ); executive dysfunction and disinhibition as measured by the frontal systems behavioral scale (FrSBe); and depression as measured by the BDI. Additional covariates were not added due to the relatively small sample size in this dataset.

### Relevance to primary anxiety disorders

Three of the seven datasets recorded presence of baseline anxiety disorders using a standardized psychiatric diagnostic tool. Anxiety disorders were defined as generalized anxiety disorder, panic disorder, social anxiety disorder, and phobias. Obsessive-compulsive disorder and posttraumatic stress disorder were not classified as anxiety disorders, as per DSM-5 guidelines. In the OPT-TMS dataset and the St. Louis DBS dataset, diagnosis was assessed using the Structured Clinical Interview for the DSM. In the THREE-D dataset, diagnosis was assessed using the Mini International Neuropsychiatric Interview.

To assess the relevance of diagnosis, we classified participants in these three datasets into two groups: those with anxiety disorders and those without. In each group within each dataset, we predicted treatment-induced anxiety change (controlling for depression) using baseline stimulation site connectivity to an anxiety circuit derived from the remaining datasets. Across all three datasets, we combined these results into a weighted mean correlation.

## Data and materials availability:

The pipeline used to prepare the functional connectivity data is available at https://github.com/bchcohenlab/BIDS_to_CBIG_fMRI_Preproc2016. Statistical analyses were performed in MATLAB R2021b. Custom MATLAB scripts for spatial permutation testing are available on our website: https://siddiqi.bwh.harvard.edu/data-code/.

The functional connectivity data employed in this study are available online through the Harvard Dataverse at https://doi.org/10.7910/DVN/ILXIKS. The final anxiety circuit will be made available on our website upon completion of peer review: https://siddiqi.bwh.harvard.edu/data-code/.

Due to the potentially identifiable nature of clinical neuroimaging data, individual lesion and stimulation data are not publicly available, but may be shared with an institutional data use agreement and institutional review board approval. These data were acquired at multiple different institutions around the world – sharing of any given dataset will be subject to the policies of the institution and the laws of the country where the dataset was collected.

## Figures and Tables

**Table 1:** Seven datasets (total n = 936) were included in this analysis across various stimulation sites, study settings, sample sizes, outcome metrics, patient populations, and localization approaches. Modalities were classified into four categories: TMS without individualized fMRI, TMS with individualized fMRI, lesions, and DBS. The modalities were classified into three categories, including DBS, TMS, and lesions. Neuronav = neuronavigation, pTBI = penetrating traumatic brain injury, STN = subthalamic nucleus, U. = University, PD = Parkinson’s disease, BDI = Beck Depression Inventory, HAMD = Hamilton Depression Rating Scale, NBRS = Neurobehavioral Rating Scale, HADS = Hospital Anxiety and Depression Scale, BSI-A = Brief Symptom Inventory for Anxiety, STAI = Spielberger Trait Anxiety Inventory, fcMRI = functional connectivity MRI, MUSC = Medical University of South Carolina, NYPH = New York Presbyterian Hospital, UW = University of Washington Medical Center, CAMH = Center for Addiction and Mental Health (Toronto), UHN = University Health Network (Toronto), UBC = University of British Columbia.

Modality	Dataset identifier	Institution of data collection and ethics approval	Setting	*n*	Anxiety Measure	Depression Measure	Diagnosis	Analysis localization approach	Age, mean (SD)	Sex	Anxiety ~ Depression correlation
Scalp-based TMS target	Boston^17^	Beth Israel Deaconess Medical Center	Naturalistic	30	BDI clusters	BDI clusters	MDD	Retrospective Neuronav	53 (10)	33% M, 67% F	r = 0.26
Scalp-based TMS target	OPT-TMS^18^	MUSC, NYPH, Emory U., UW	Multi-center trial	81	HAMD	HAMD clusters	MDD	MRI fiducial marker	47 (11)	57% F, 43% M	r = 0.57
Lesions (pTBI)	VHIS^19^	US Army	Cross-sectional	181	NBRS	NBRS	pTBI	CT (chronic)	58 (3)	100% M	r = 0.54
Lesions (stroke)	Cologne^20^	U. Leipzig Medical Center	Cross-sectional	270	HADS	HADS	Stroke	MRI	66 (12)	61% M, 39% F	r = 0.58
MRI-guided TMS	THREE-D^21,22^	CAMH, UHN, UBC	Multi-center trial	300	BSI-A	HAMD	MDD	Individualized fcMRI	43 (12)	59% F, 41% M	r = 0.52
DBS (STN)	Pittsburgh^23^	U. Pittsburgh Medical Center	Cohort	36	Zung	BDI	PD	CT and MRI (postop)	68 (8)	71% M, 29% F	r = 0.45
DBS (STN)	St. Louis^24^	Barnes-Jewish Hospital, Washington U.	Cohort	38	STAI	BDI	PD	CT (postop)	63 (8)	51% F, 49% M	r = 0.22
